# Changes in the upper ocean mixed layer and phytoplankton productivity along the West Antarctic Peninsula

**DOI:** 10.1098/rsta.2017.0173

**Published:** 2018-05-14

**Authors:** Oscar Schofield, Michael Brown, Josh Kohut, Schuyler Nardelli, Grace Saba, Nicole Waite, Hugh Ducklow

**Affiliations:** 1 Rutgers University's Center for Ocean Observing Leadership (RU COOL), Department of Marine and Coastal Sciences, School of Environmental and Biological Sciences, Rutgers University, New Brunswick, NJ 80901, USA; 2 Lamont-Doherty Earth Observatory, Columbia University, Palisades, NY 10964, USA

**Keywords:** West Antarctic Peninsula, chlorophyll *a*, phytoplankton, upper mixed layer depth

## Abstract

The West Antarctic Peninsula (WAP) has experienced significant change over the last 50 years. Using a 24 year spatial time series collected by the Palmer Long Term Ecological Research programme, we assessed long-term patterns in the sea ice, upper mixed layer depth (MLD) and phytoplankton productivity. The number of sea ice days steadily declined from the 1980s until a recent reversal that began in 2008. Results show regional differences between the northern and southern regions sampled during regional ship surveys conducted each austral summer. In the southern WAP, upper ocean MLD has shallowed by a factor of 2. Associated with the shallower mixed layer is enhanced phytoplankton carbon fixation. In the north, significant interannual variability resulted in the mixed layer showing no trended change over time and there was no significant increase in the phytoplankton productivity. Associated with the recent increases in sea ice there has been an increase in the photosynthetic efficiency (chlorophyll *a*-normalized carbon fixation) in the northern and southern regions of the WAP. We hypothesize the increase in sea ice results in increased micronutrient delivery to the continental shelf which in turn leads to enhanced photosynthetic performance.

This article is part of the theme issue ‘The marine system of the West Antarctic Peninsula: status and strategy for progress in a region of rapid change’.

## 1. Introduction

The West Antarctic Peninsula (WAP) is a highly productive marine ecosystem characterized by large phytoplankton blooms [1–4]. The high productivity supports a productive food web tightly coupled to seasonal phytoplankton dynamics suggesting bottom-up control of the ecosystem [5]. Seasonal phytoplankton activity peaks when solar illumination increases in the summer when ice has melted and the ocean surface is exposed [6–7]. The physical factors that influence upper ocean stability during spring are critical to explaining the dynamics of phytoplankton blooms on the WAP [8–11]. In high ice years, seasonal melt results in a stable water column that retains phytoplankton in a shallower surface layer, where light conditions are favourable for phytoplankton growth [9,12,13]. In low ice years, enhanced wind mixing and deeper mixed layers result in phytoplankton experiencing lower light levels and overall primary productivity is reduced. The argument for light regulation of phytoplankton growth is also supported by the high concentrations of macro- [14] and micronutrients [15] in coastal areas and the inner continental shelf of the WAP; however, low concentrations offshore suggest the possibility for micronutrient limitation in this region [15].

The WAP is experiencing significant change. It is one of the most rapidly winter warming regions on Earth, with annual mean atmospheric warming of 3.7°C/century and more than 1°C surface warming ocean during last 50 years [16–21]. Despite the observed changes, current temperatures remain within natural climate variability for this region and so change cannot yet be ascribed to anthropogenic driven change [22]. However, the rapid change makes it a valuable model system to study how future changes might ripple through polar ecosystems [23]. Associated with atmospheric warming are numerous observations of changes in the sea ice-, upper ocean heat- and freshwater dynamics [24]. For example, near Palmer Station in the central WAP region, the annual ice season has declined by 92 days over the last 35 years [24,25]; however, since 2008 there have been rebounds in the amount of winter sea ice around Palmer Station [26]. The long-term trends in sea ice have been associated with a myriad of effects ranging from wind-driven circulation to upwelling of warm upper circumpolar deep water (UCDW) on the continental shelf [27,28]. These processes are influenced by the Southern Annular Mode (SAM) and the El Niño-Southern Oscillation (ENSO) [25,29]. Changes are believed to underlie long-term change in the phytoplankton biomass [30]. In the northern WAP, long-term declines in phytoplankton biomass have been associated with reduced water column stratification due to more vigorous wind-mixing and increased clouds decreasing the absolute light flux [30]. By contrast, in the southern WAP, there has been a long-term increase in phytoplankton biomass as the system transitions to more open water. This is hypothesized to increase the available light [30], consistent with the overall hypothesis that primary productivity in this region is light limited. Long-term studies of the dynamics in the upper mixed layer depth (MLD) are, therefore, critical to understanding dynamics in ecosystem.

Regional decadal studies of the dynamics in the upper mixed layer and its relationship to the phytoplankton in the Southern Ocean have been limited to local sampling from shore-based field stations [10,26,31,32]. We use the long-term dataset collected by the PALmer Antarctica Long Term Ecosystem Research (PAL) programme's annual ship surveys of coastal regions, the continental shelf and continental slope. For this study, we assess long-term trends in the upper water column stability and phytoplankton dynamics. Our results show that the upper MLD in the southern regions of the WAP has declined significantly over the last two decades. These changes have significant implications for the ecosystem and have resulted in an increase in biomass-normalized phytoplankton productivity.

## 2. Material and methods

### (a) Site description

**Figure 1.**
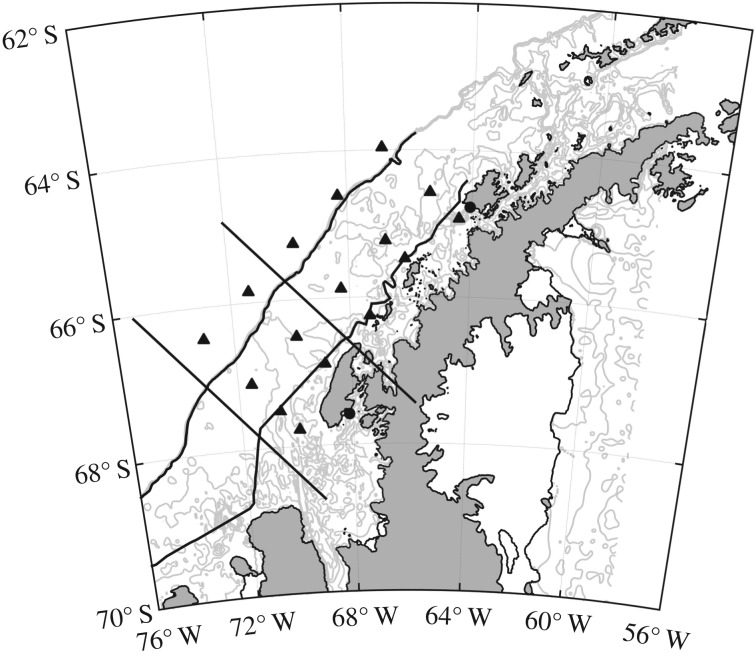
Map of the study sites along the West Antarctic Peninsula sampled by the Palmer Long Term Ecological Research (LTER) programme. The triangles indicate stations occupied over the 25 year time series. The black lines separate the North and South regions used in this study. The circles represent laboratory-based stations. In the north is the United States Palmer Station and in the south the United Kingdom Rothera Station.

The PAL programme was initiated in 1991 to study how annual sea ice variability structures the ecology of the West Antarctic Peninsula (WAP). A major component of PAL is an annual ship survey conducted each austral summer in the month of January. The cruise conducts a series of cross shelf transects that span a north–south gradient. Over the course of the programme, there have been adjustments to the sampling grid in response to changes in the sea ice distributions over time [24]. For this analysis we use a subset of the stations that were sampled across the full time series. We split the sampling grid into northern (600, 500, 400 lines) and southern (300, 200 lines) regions following the analysis of Steinberg *et al*. [33] (figure 1). Seasonal weather data (air temperature, pressure, wind speed, wind direction, precipitation, sky cover) were collected at Palmer Station where the time series combined manual observations (1989–2003) and PALMOS automatic weather station measurements (2003–2017). All PAL data are publicly available through the Palmer Long Term Ecological Research (LTER) data system (http://pal.lternet.edu/data).

### (b) Data treatment

Sea ice was characterized using Version 2 of the Bootstrap sea ice concentrations from Nimbus-7 SMMR and DMSP SSM/I that are referenced to daily tie-points consistent with the AMSR-E Bootstrap algorithm. GSFC Bootstrap time series data (version 2.0) for January 1979 to October 2010, supplemented by preliminary Near-Real-Time-Sea-Ice (NRTSI) data (using the NASA Team algorithm) for November–December 2010. See Stammerjohn *et al*. [25] for further details. For all correlations we used least-squares linear regression.

During the cruise, each station was sampled by a rosette equipped with a SeaBird CTD system and sampling bottles from 1998 to the present. Prior to 1998 (1993–1998), temperature and conductivity data were collected with an internally recording Seabird SEACAT system integrated into the Bio-Optical Profiling System [34]. The CTD sensors were pre- and post-cruise calibrated by SeaBird. Standard post-cruise calibration corrections were applied to the data, along with standard data reduction. Seawater samples were collected at discrete depths with Niskin bottles. The seawater was immediately filtered (1–2 l) onto 25 mm GF/F filters, wrapped in foil and frozen at −80°C for fluorometric phytoplankton chlorophyll *a* (Chl *a*) analysis (mg Chl *a *m^−3^). For this study, the Chl *a* concentrations were integrated in the upper 100 m of the water column and then averaged for the stations within the northern or southern domain. Primary productivity rates were measured by the uptake of radioactive sodium bicarbonate. In borosilicate flasks, 100 ml aliquots of the sea water, collected by the CTD at selected depths (at minimum five depths), were inoculated with nominally 1 µCi of NaH^14^CO_3_ per bottle. Integrated rates were calculated for the upper 100 m of the water column. The borosilicate bottles were incubated for 24 h with bottles screened to *in situ* light levels. Given the bottles were not ultraviolet transparent, these photosynthesis rates are likely to be upper limit estimates. Samples were incubated in an outdoor deck incubator. After incubation, samples were filtered onto GF/F filters, washed with 10% HCl, dried and counted in a scintillation counter.

To calculate the seasonal MLD, we used the approach described in Carvalho *et al*. [35]. For each profile, surface MLD is estimated by finding the depth of the maximum water column buoyancy frequency, or max(*N*^2^). This method is focused on the water column vertical structure, a quality index (QI) filter is also applied to identify water column profiles without significant stratification. For this we used QI (equation (2.1)) developed by Lorbacher *et al*. [36] to evaluate individual MLD calculations against water column density and filter out profiles where MLD could not be resolved. The QI is calculated as
2.1
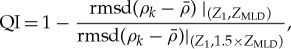

where *ρ_k_* is the density at a given depth (*k*), *Z*_1_ is the first layer near the surface and rmsd() denotes the standard deviation from the vertical mean 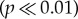 and 1.05 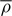 from *Z_1_* either to the MLD or 1.5×MLD. This index evaluates the quality of the MLD computation. Using this, MLDs can be characterized into estimates determined with certainty (QI > 0.8), determined but with some uncertainty (0.5 < QI < 0.8) or not determined (QI < 0.5). Following the thresholds set by Lorbacher *et al*. [36], for the analyses presented in this study, a QI of 0.5 was used to reasonably warrant a calculation of MLD. This determination of MLD is based on the principle that there is a near-surface layer characterized by quasi-homogeneous properties and where the standard deviation of the property within this layer is close to zero. This method does not consider the strength of stratification, just homogeneity of the surface layer present. Therefore, by definition the MLD estimate is close to the lower boundary of that vertically uniform layer. The method has been validated for locations across the Southern Ocean (WAP, Amundsen, Ross Sea) and its ecological relevance was confirmed against independent chlorophyll data [35]. The QI threshold flagged 19% of the profiles (161 out of the 825 profiles) as not having a MLD being reliably determined. To assess the impact on the seasonal MLDs of omitting the profiles, we compared using all profiles to those with a QI ≥ 0.5 (table 1). Results showed they provided similar MLD values. Additionally, the correlation slopes between the annual mean MLD for all profiles versus those with a QI ≥ 0.5 provided highly significant linear relationships. The correlation slopes were 0.89 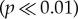 and 1.05 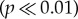 for the north and south respectively.

**Table 1.** Calculated upper MLDs for all profiles and those profiles with a QI greater than 0.5 for the northern and southern regions of the LTER study area.

variable dataset	mean	STD
North sector MLD (all profiles)	34	23
North sector MLD (QI ≥ 0.5)	33	25
South sector MLD (all profiles)	36	20
South sector MLD (QI ≥ 0.5)	36	21

## 3. Results

### (a) Sea ice, mixed layer depth and wind

**Figure 2.**
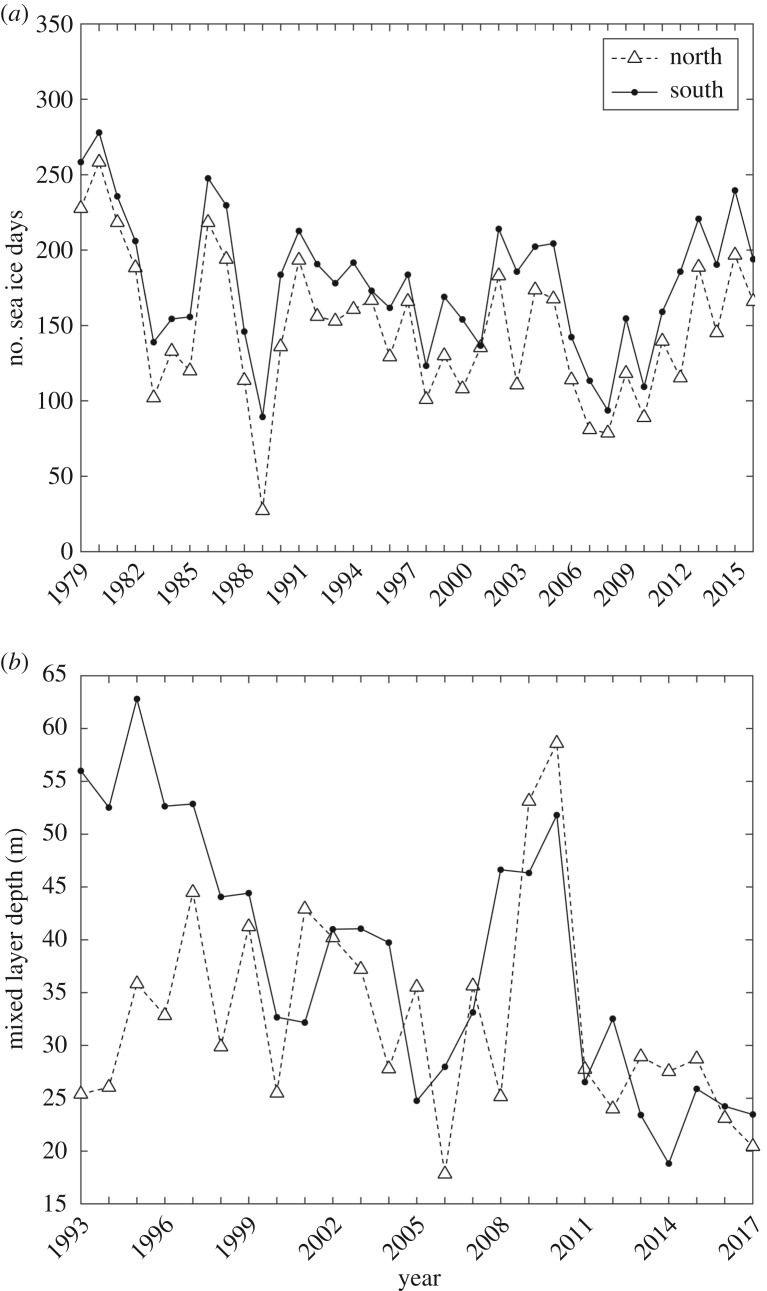
Times series data collected during the Palmer LTER programme for averaged data for the northern (open triangle and dotted lines) and southern regions (solid black line and solid circles) collected during cruises conducted each January. (*a*) The times series of the number of sea ice days for both the northern and southern regions. (*b*) The MLD, calculated according to Carvalho *et al*. [35], for the northern and southern regions.

Sea ice concentrations showed significant interannual variability in the WAP (figure 2*a*). There was a positive correlation between the number of sea ice days in the north and south over time (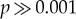, *R*^2^ = 0.89). On average there are approximately 30 fewer sea ice days in the north compared to the south. The number of sea ice days significantly declined from 1979 through 2008 in both the north (*p* = 0.01) and south (*p* = 0.01) with declines of 44 and 41 days respectively (figure 2*a*). These changes appeared to be largely related to a later sea ice advance in the north. In the south the declines in sea ice days were associated with a later seasonal advance and an earlier retreat in austral spring. Since 2008, sea ice days have increased significantly in the north (*p* = 0.03) and south (*p* = 0.03) almost recovering to the sea concentrations observed in the mid-1980s. In the north and south the increase was associated with an earlier sea ice advance in autumn.

**Figure 3.**
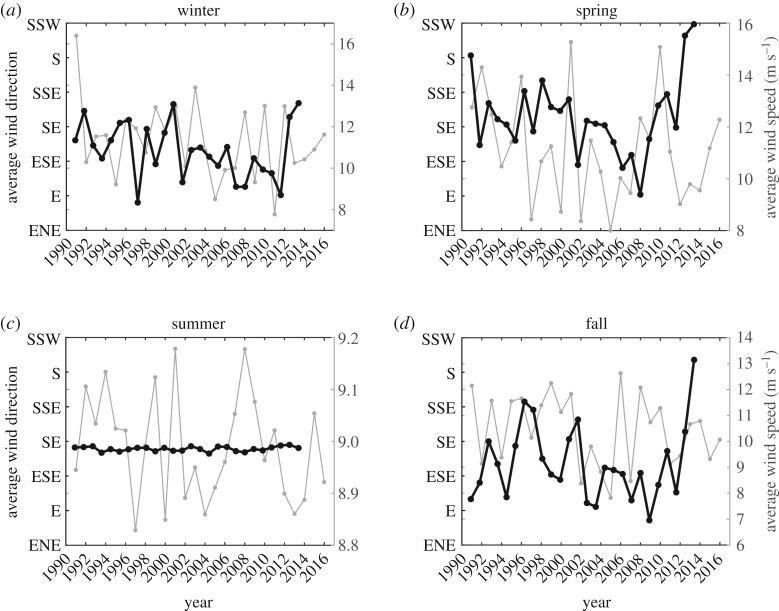
Seasonally averaged wind data collected at the United States Palmer Station since 1990. The data for the winter (*a*), spring (*b*), summer (*c*) and fall (*d*) are presented. The right axis provides the seasonal average wind direction (black line) while the left axis (grey line) shows average wind speed (m s^−1^).

Seasonally averaged time series of the wind speed and direction at Palmer Station are provided in figure 3. Wind speed showed significant variability over all seasons with no significant trended change in any of the seasons given the high interannual variability (figure 3). Wind speeds were greatest in the fall, winter and spring with seasonally averaged wind velocities ranging between 8 and 16 m s^−1^. The summer season showed lower variability and magnitude in wind velocity ranging from 8.8 and 9.2 m s^−1^ (figure 3*c*). Wind direction showed fall and spring shifts from SE to SSW and S, respectively from 1990 to 2009 (figure 3*b,d*).

There were significant differences between MLDs in the northern and southern regions of the WAP (figure 2*b*). In both regions there was significant interannual variability in the MLD which could increase/decrease by a factor of 2 between years. For the northern (*p* = 0.06) and southern (*p* = 0.03) regions there were negative correlations between the number of sea ice days and MLD, with large sea ice years associated with shallower seasonal MLDs. There were some notable differences between the northern and southern regions. In the northern region there was no significant trend over time in the MLD (*p* = 0.40), and mixed depths ranged from 17 to 60 m (figure 2*b*); however, the majority of the years the MLDs ranged between 25 and 45 m (figure 2*b*). In contrast, MLDs in the southern region showed declines over 25 years (figure 2*b*). In the early 1990s MLDs were typically deeper than 50 m and have since declined by 50% to 25 m. The declining trend in the southern upper MLD over time was highly significant (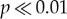, *R*^2^ = 0.58) and there was a negative correlation (*p* = 0.03) between sea ice retreat and MLD.

**Figure 4.**
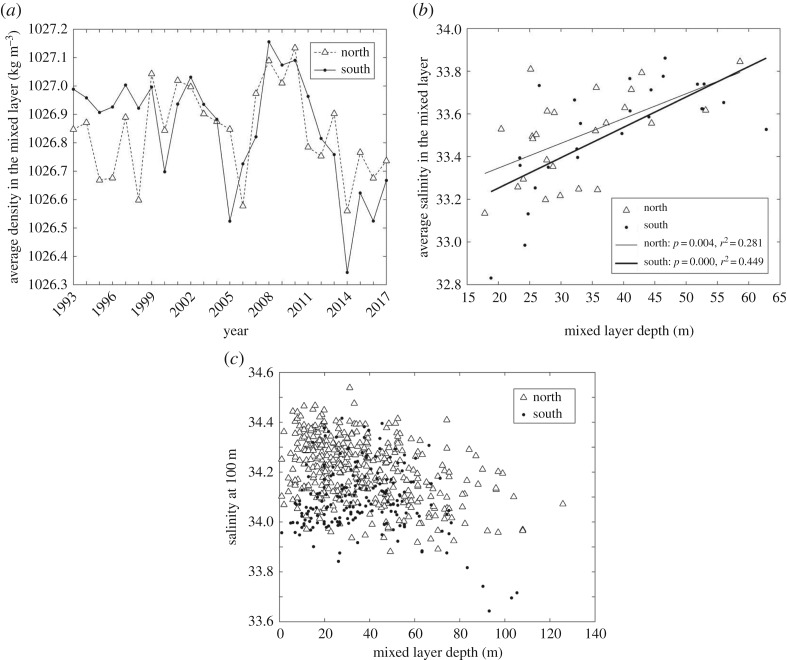
(*a*) Time series for the average density in the upper mixed layer. The data for the northern (open triangle and dotted lines) and southern regions (solid black line and solid circles) collected during cruises conducted each January. (*b*) The correlation between average salinity in the mixed layer for the MLD. The triangles represent the northern region and southern regions are represented in solid circles. (*c*) The relationship between the salinity at 100 m and the upper MLD. There are no trended declines in the salinities at 100 m until upper MLD are deeper than 60 m.

Seawater density showed declining values (1027 to 1026.4) in the upper MLD in the southern region over time (figure 4*a*). The changes in density were largely driven by strong declines occurring after 2008, which mirrored declining upper ocean salinities and temperatures. The timing of these changes was coincident with the reversal in sea ice loss (figure 2*a*). There was no significant decline in the density for the northern region (figure 4*a*). The MLDs in the north (*p* < 0.01, *R*^2^ = 0.28) and south (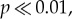 *R*^2^ = 0.45) were positively correlated with the salinity in the upper water column (figure 4*b*). This suggests that sea ice melt was a dominant factor determining the depth of the upper MLD. Examination of the deep salinities at 100 metres, showed values were stable whenever the MLD was 60 metres or shallower suggesting that wind-driven mixing was minimal (figure 4*c*). The salinities showed declines when the MLD was greater the 60 m, which represented 13% of the profiles in the north and 10% of the profiles in the south.

### (b) Phytoplankton biomass

**Figure 5.**
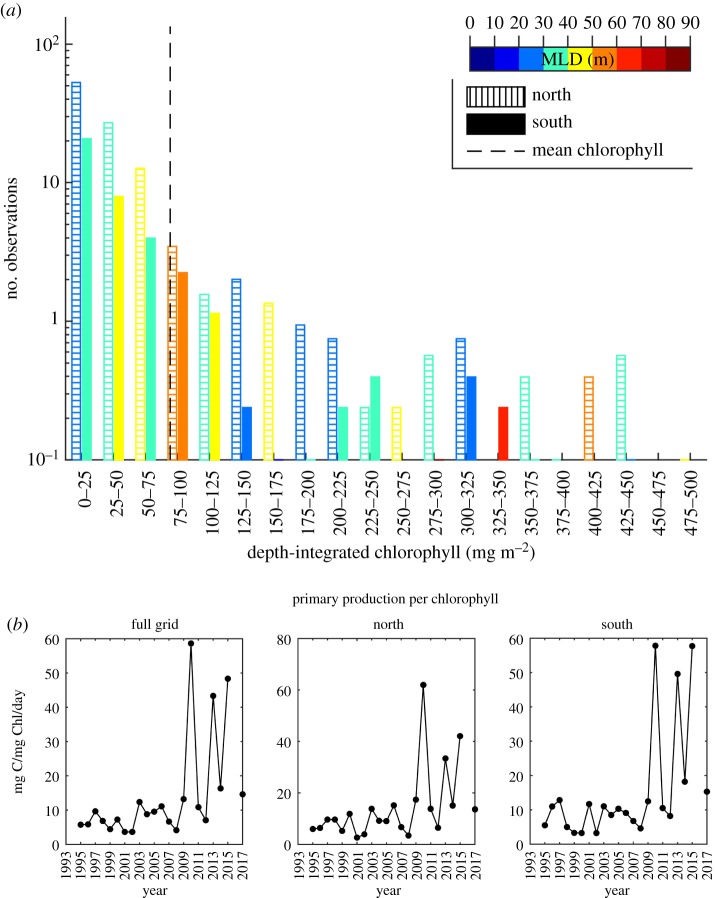
(*a*) The histogram between the number observations and depth-integrated chlorophyll *a* (Chl *a *m^−2^). The colour on the bars represents the depth of the depth of the MLD. The solid coloured bars represent the data collected in the southern region while the stripped open columns represent the northern region in this study. (*b*) Time series of the water column daily primary productivity normalized to chlorophyll *a* (mg C mg Chl *a*^−1^ day^−1^).

The northern and southern regions of the WAP are highly productive. Water column integrated chlorophyll often exceeded 200 mg Chl *a* m^−2^ in both the northern and southern regions (figure 5*a*). The average Chl *a* over the WAP full sampling grid was 79 mg m^−2^. The mean Chl *a* in the north was 82 mg Chl *a* m^−2^ compared to 71 mg Chl *a* m^−2^ in the southern region. The Chl *a* stocks that were higher than the mean were heavily weighted to MLDs shallower than 40 metres (figure 5*a*). There was high interannual variability in the depth-integrated Chl *a* and no significant correlation with MLD. Primary productivity also did not show any significant trends over time in the north. In the southern region where the MLD had declined there was a significant negative correlation of MLD (*p* = 0.05) with volumetric primary production. There was a significant increase in the volumetric primary productivity (*p* = 0.02, *R*^2^ = 0.25) for both the north and south after 2008, with five of the eight years exhibited elevated photosynthetic rates. The chl-normalized primary productivity (mg C mg Chl *a*^−1^ day^−1^) also showed an increase of a factor of 4- and 5-fold for the north and south, respectively, after 2007 (figure 5*b*). The increases over time were highly significant for both the north (*p* = 0.02, *R*^2^ = 0.24) and the south (*p* = 0.01, *R*^2^ = 0.28). The increases in Chl *a*-normalized productivity were coincident with the increases in sea ice beginning after 2008.

## 4. Discussion

The northern regions of the peninsula show dramatic declines in sea ice and significant glacial retreat while the southern peninsula remains a cold dry polar system [24]. The regional differences in the physical system are mirrored in the biology. Long-term studies have linked changes in the phytoplankton biomass to changes in sea ice, wind and clouds [30]. This study showed that phytoplankton biomass increased in southern regions of the WAP between the mid-1980s and the early 2000s before the recent reversal (figure 2*a*). It should be noted that all data in this study were located in the southern region as defined by Montes-Hugo *et al*. [30]. Phytoplankton increases in south were hypothesized to reflect increases in light in the ocean associated with declines in ice cover [30]. Additionally, the total amount of sea ice/glacial melt in the spring helps stabilize the water with a surface layer of lower salinity water. Saba *et al.* [5] found that winters that had significant sea ice were associated with shallower and more stable seasonal upper mixed depth in the spring. High ice extent facilitates stratification in the following spring and summer via two mechanisms, (1) insulation of the water column from high winds through much of the winter and spring thus preventing the formation of a deep winter mixed layer [11,30] and (2) providing a larger volume of sea ice melt water that strengthens the density gradient in the upper water column in the following spring and summer [12]. This was confirmed at Palmer Station where the summer MLD and the length of the summer growing season were the critical factors driving the size of the summer phytoplankton blooms [26]. Our regional data support this interpretation where the low salinity water results in shallower mixed layer, alleviating light limitation. For this dataset there were no significant increases in Chl *a* over time; however, in the southern region there was a significant increase in the phytoplankton volumetric carbon fixation that has been positively correlated with the concentration *Euphausia superba* [33]. Thus clarifying the key environmental factors resulting in enhanced phytoplankton productivity, less so for Chl *a*, remains the key need to predicting future trends in the higher trophic levels. Given this our results suggests the southern WAP food web remains a prototypical short, efficient food chain dominated by high productivity rates often characterized by larger phytoplankton and zooplankton. The southern region's food webs are multivorous, with energy flows more or less equally divided between large and small producers and consumers [37]. This is in contrast to the northern regions where the food web is transitioning to a more dissipative microbial food web dominated by small phytoplankton and grazers [37].

The return of the sea ice since 2008 was associated with an increased efficiency of phytoplankton photosynthesis (figure 5*b*). The large increases might reflect two processes. One possibility is that cells reside in shallower mixed layers and thus higher light levels. Phytoplankton are capable of photoacclimating to the higher light levels by adjusting the amount of Chl *a* cell^−1^. Phytoplankton in the WAP have been shown to actively photoacclimate to changes in the depth of the mixed layer [9]. In the laboratory, cells have been shown to decrease the amount Chl *a* cell^−1^ by a factor of 2 [38] but given variable wind mixing in these waters, cell photoacclimation is likely to be dampened. This is consistent with the relatively limited variability observed in photosynthesis–irradiance curves from this region [39]. Therefore, while photoacclimation is present, it is unlikely to account for the factor of 4 increases in Chl *a*-normalized productivity. It has been well established that cells overcoming nutrient limitation will show increased photosynthetic efficiencies [40]. In the WAP, iron concentrations vary widely across the region [15]. Dissolved and particulate Fe were high in coastal waters (up to 8 and 42 nmol kg^−1^, respectively). In contrast, very low Fe concentrations (<0.1 nmol kg^−1^) are seen in mid- to outer-shelf surface waters, suggesting possible Fe limitation of primary production on the shelf. Sea ice and dust inputs of Fe appear to be minor, although their relative importance increased with distance from shore due to the larger near-shore sources [15]. Overall, the interannual distribution of Fe was most closely correlated to that of meteoric water (glacial melt and precipitation). Although the Fe concentrations and relative contributions of dissolved and particulate Fe attributed to meltwater were variable throughout the sampling region [15], increasing glacial meltwater flux can be expected to increase the delivery of Fe to surface waters along coastal and across the shelf of the WAP. This combined with recent increases in sea ice, increasing the importance of this source of iron, is likely to result in increased photosynthetic performance.

## 5. Conclusion

The number of sea ice days on the WAP had been steadily declining since the 1980s until a recent reversal. In the southern WAP, the upper ocean MLD has declined by a factor of 2. Associated with the shallower MLDs were enhanced phytoplankton carbon fixation rates. In the northern region, there was significant interannual variability in the MLD and there was no trended change over time. There was no significant increase in the phytoplankton productivity in the north. Associated with the recent increases in sea ice over the last decade, there has been an increase in the photosynthetic efficiency (chlorophyll *a*-normalized carbon fixation) in the WAP. We hypothesize the increase in sea ice results in an increase in micronutrients on the continental shelf that in turn leads to enhanced photosynthetic performance. Results show the close relationship between the biology and the sea ice on the WAP. Continued temperature changes along the WAP will result in changes in phytoplankton communities which has significant ramifications for the food web.

## Data accessibility

Primary data used in the present study can be assessed at the Palmer LTER Datazoo (http://pal.lternet.edu/data).
